# Emergency Department Utilization for Patients Receiving Immune Checkpoint Inhibitors: A Retrospective Analysis of Identification and Outcomes for Those Presenting for Immune-Related Adverse Events

**DOI:** 10.3390/curroncol28010007

**Published:** 2020-12-02

**Authors:** Ryan Holstead, Adi Kartolo, Tara Baetz

**Affiliations:** Cancer Centre of Southeastern Ontario, Department of Oncology, Queen’s University, Kingston, ON K7L 2V7, Canada; ryangordon.holstead@kingstonhsc.ca (R.H.); BaskoroAdi.Kartolo@kingstonhsc.ca (A.K.)

**Keywords:** immune checkpoint inhibitors, immunotherapy, immune related adverse events, emergency department

## Abstract

Background: Immune-related adverse events (iRAEs) are known complications of immune checkpoint inhibitors (ICIs). Early identification and management leads to improved morbidity and mortality. This study seeks to address our center’s experience with iRAEs in the emergency department (ED). Methods: We performed a retrospective review of patients treated with ICIs in 2018 and 2019 for any indication. All diagnoses of iRAEs were recorded. For all patients who presented to the ED following administration of an ICI, we assessed whether the presenting symptoms were eventually diagnosed as an iRAE. We assessed disposition, time to initiation of corticosteroids and outcomes in these patients. Results: 351 evaluable patients were treated with an ICI, 129 patients (37%) had at least one presentation to the ED, 17 of whom presented with symptoms due to a new iRAE. New iRAE diagnoses were broad, occurred after median 2 cycles, majority irAEs were grade 3 or higher (70.6%), and two patients died due to toxicity. Twelve patients were admitted to the hospital during initial presentation or at follow-up, four required ICU care. All patients required immunosuppressive therapy, and only three were later re-challenged with an ICI. Of the patients who were admitted to the hospital, median time to first dose of corticosteroid was 30.5 h (range 1–269 h). Conclusions: Patients on ICI have a significant risk of requiring an ED visit. A notable proportion of iRAEs have their first presentation at the ED and often can present in a very nonspecific manner. A standardized approach in the ED at the time of presentation may lead to improved identification and management of these patients.

## 1. Introduction

Immune checkpoint inhibitors (ICIs) have led to a paradigm shift in the practice of medical oncology. Inhibition of the programmed death pathway (PD-1/PD-L1 inhibitors; e.g., pembrolizumab, nivolumab, atezolizumab, avelumab, durvalumab, cemiplimab) or cytotoxic T-lymphocyte-associated protein 4 (CTLA-4 inhibitors; e.g., ipilimumab and tremelimumab) have revolutionized the approach to many types of cancer, with a growing number of indications annually [1,2,3,4,5,6]. By “activating” a patient’s immune system, ICI mechanism of activity is unique when compared to cytotoxic chemotherapies or other molecularly targeted agents [7].

Toxicities from ICI can arise when the immune system inappropriately acts on noncancerous cells [8]. These immune-related adverse events (irAEs) have variable timing of onset, presenting symptoms, severity, with the potential to affect any organ system. Management of toxicity requires timely identification and initiation of therapy, which often includes systemic corticosteroids (CS) [9]. A delay in CS initiation can lead to a worsening severity of toxicity. Severe toxicities may be irreversible, lethal, requiring intensive immunosuppression, and may preclude further cancer treatment with ICI therapy [10].

Providers from various medical subspecialties have assisted with development of standardized approaches to the initial treatment and monitoring of irAEs, dependent on severity grade [9,10]. Much of this management borrows from experience with various autoimmune diseases, which have shown efficacy for irAEs due to ICIs. Cancer care organizations have provided guidelines to aid the prescribing oncologist with monitoring patients on therapy and approaching care as toxicities arise. Some cancer centers provide immune therapy education classes to teach patients on what symptoms may require an expedited assessment by their provider [11].

Invariably, patients may develop symptoms while on ICI and subsequently present to the emergency department (ED) for assessment. Providers in the emergency department may not be aware that the patient is on ICI therapy or may not be aware of the potential for irAEs. Patients with advanced cancer may present to the ED for numerous reasons including disease progression, infections, pain crisis. This places a challenge on identifying a first presentation of an irAE among other potential causes of generalized fatigue or pain [12,13,14].

As the number of patients receiving ICI is increasing, it is important to understand the impact that these patients have on the ED. In this study, we reviewed patients receiving ICI therapy from the Cancer Center of Southeastern Ontario, which services a regional population of 550,000. We sought to seek outcomes of the patients who had their first presentation of an irAE in the ED and compared them to the toxicities that arose in the total population of patients receiving ICI within our center.

## 2. Methods

We conducted a retrospective review of patients treated with immune checkpoint inhibitors at the Cancer Center of Southeastern Ontario (CCSEO) at the Kingston Health Sciences Centre in Kingston, Ontario, Canada, in 2018 and 2019. This is an academic tertiary care cancer center associated with Queen’s University. This study was approved by the research ethics board at Queen’s University. All of the ED visits for ICI patients presenting to one of the two EDs associated with the Kingston Health Sciences Centre were captured. Patients who were on combination chemotherapy/immunotherapy regimens were included. Patients were excluded if they were on a clinical trial that remained blinded at the time of data extraction and if they were lost to follow-up following the initial treatment with ICI therapy.

All ED visits that occurred following at least one cycle of ICI were reviewed. If a patient had multiple ED visits, the first that was related to a new irAE was analysed. If the patient did not have a new irAE ever diagnosed in the ED, the first for complications of a previously diagnosed irAE were included and if a patient never had a visit for irAE-related symptoms, their first visit was analysed. Toxicity diagnosis was confirmed by analysis of hospitalization course, or outpatient oncology follow-up documentation. For the analysed ED visit, we recorded presenting symptoms, number of ICI cycles prior to presentation, diagnosis made by ED provider, patient disposition. If the presentation was confirmed to be the first presentation of a new irAE, we recorded the time to oncology consult/assessment, time to CS initiation, toxicity grade, length of hospitalization, 30 day ICU admission and whether ICI was ever restarted.

For all patients who received ICI, we extracted demographic data, tumor site, drug used, indication for therapy, and all irAEs (including toxicity grade, symptom resolution, and whether ICI was restarted from charts. All toxicities were graded using the Common Terminology Criteria for Adverse Events (CTCAE) v5.0 [15]. Data from emergency presentations were extracted, including presenting time to initiation of steroid treatment and disposition. Descriptive statistics were used to define populations of patients with irAE as well as time to initiation of steroids, rates of toxicity, and outcomes for all patients with irAEs. We compared severity of irAEs diagnosed in ED to the total population. We also compared rates of ED visits and rate of irAE in patients receiving doublet ICI (dICI) therapy compared to single-agent regimens.

## 3. Results

From 2018 to 2019, 351 patients were identified who had at least one dose of standard of care ICI treatment and for whom we had follow-up data. The demographics and indications are summarized in Table 1; 111 (32%) of these patients would develop at least one irAE during treatment, 28% of these were grade 3 or higher, 41 of the patients received at least one cycle of a doublet immunotherapy (dICI) regimen, with 21 (48.8%) developing irAEs of any grade.

**Figure 1:**
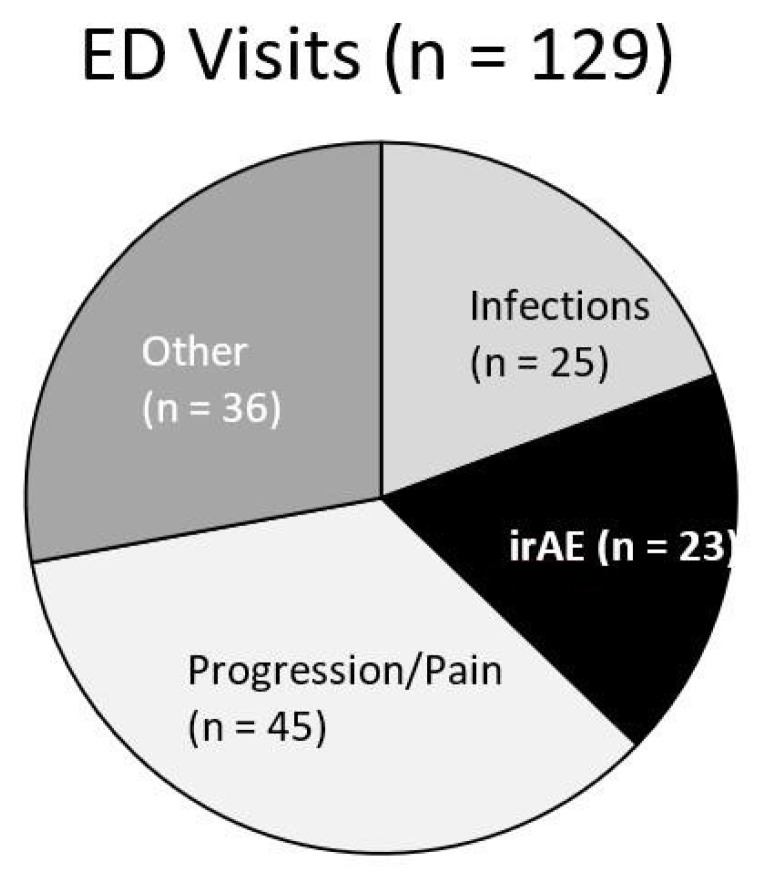
Pie chart for patients who received immune checkpoint inhibitors (ICI) with subsequent presentation to ED and proportion of indications for visit. Visits categorized as irAE (black) include first presentation of a new toxicity as well as presentation for complications of an already known irAE.

**Figure 2:**
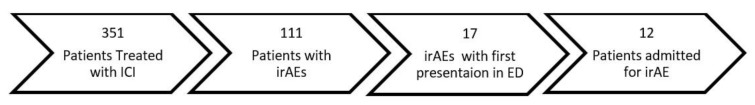
Flow diagram demonstrating the number of patients receiving immune checkpoint inhibitors (ICIs), the number of immune-related adverse events (irAEs), the number of patient with an irAE first diagnosed in the ED and the number of patients admitted as a result.

**Figure 3:**
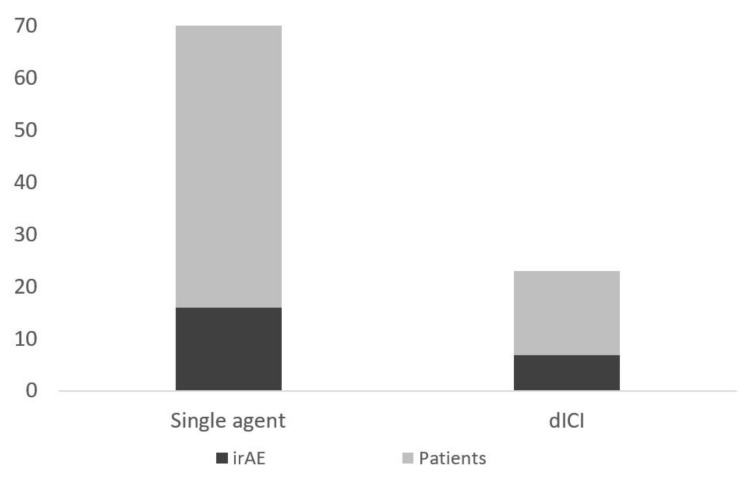
Number of patients presenting to ED (total height) and those with an ED visit for symptoms due to an irAE (darker shaded bar) separated by those receiving single-agent and dICI regimens.

Of the entire cohort, 129 (37%) patients had at least one presentation to the emergency department. Twenty-three patients had an ED visit related to irAEs (18%), 25 for infections (14%), 45 for cancer progression or pain (35%), and 36 for other reasons (28%) (Figure 1). Of the patients with an ED presentation, 17 (13.2%) would be diagnosed with a new irAE related to their presentation (Figure 2). Twenty-three of patients receiving dICI had at least one ED presentation and 7 of these patients had ED visits related to an irAE, all of which were the first presentations of a new toxicity (Figure 3).

Patients with an ED irAE presentation were a median of 1 month from initial treatment and after a median of 2 cycles of treatment (range 1–24) (Table 2). Presenting symptoms varied but were mostly nonspecific such as fatigue. The median time to initiation of steroids was 30.5 h with significant delays seen in patients that were not identified as an immunotherapy patient or were not considered to have an irAE as a cause of their nonspecific symptoms. Patients presenting in ED were diagnosed with a broad range of irAEs and many (7 patients) subsequently were diagnosed with multiple irAEs. Twelve (70.6%) of these patients required admission to hospital for their irAE, and two patients subsequently died of toxicity.

## 4. Discussion

In this patient population, approximately one-third of patients receiving ICI therapy had at least one presentation to the ED and those presenting with a new irAE had high-grade toxicity and often had prolonged hospitalizations. Most of the patients presenting with a new irAE had generalized symptoms, which could resemble infection or progression and have potentially delayed identification and corticosteroid initiation. Analysis of time to oncology consultation, disposition from the emergency department, or admitting service did not associate with differences in timing of corticosteroid initiation. The authors of this study believe a “door-to-steroid” time of <12 h from ED presentation is a reasonable goal that may improve patient outcomes. Understanding the source of CS delay can provide clues on a pathway to reduce this time.

Two patients did not receive corticosteroids until over 10 days from the initial presentation. One of these patients initially presented with back pain, days later developing dyspnea leading to pneumonitis diagnosis. The other patient presented with a fall, and was admitted for the medical service for failure to thrive, attributed to poor appetite and hyponatremia. Despite correction of metabolic abnormalities and nutrition, his clinical status worsened, leading to neurology, medical oncology consultation and the diagnosis of encephalitis. Both patients recovered after corticosteroid initiation, although the latter had a prolonged recovery process. The grade 5 toxicities each had a generalized presentation along with delayed corticosteroid initiation. One of the patients was admitted to medical oncology with hypotension, nausea, vomiting presumed secondary to gastroenteritis, however later developed transaminitis prompting initiation of corticosteroids. The other patient was admitted to cardiology for presumed myocardial infarction with the diagnosis of myocarditis considered after an unremarkable catheterization.

Although our total population have toxicity rates comparable to previous trial results [1,5,11], many of the patients presenting to ED with a new irAE had toxicities that are less commonly reported. Colitis, most commonly reported serious irAE (grade 3 or greater) in previous studies, was only diagnosed in two of the ED patients [16]. Previous emergency medicine publications have identified rash and diarrhea as the most common toxicities presenting to the ED [12,13,14,17], only recorded in two patients. One possible reason for this discrepancy is that the patient or prescriber may be more likely to recognize these symptoms as related to an irAE and care may have been initiated as an outpatient or with a direct admission, circumventing the ED. Our center provides ICI education courses to all patients on therapy where the most common toxicities are discussed. This finding highlights the importance of increased awareness regarding diversity in irAE presentation and the changing landscape at different points of care.

To improve upon detection and management of irAEs, the CCSEO began to provide “ICI cards” to patients on therapy, adapted from card templates published by medical oncology organizations [18,19], which identify the drug the patient is receiving, the prescribing oncologist, potential irAEs, and recommend blood work and medical oncology consultation. We have developed an order set and protocol to standardize the approach to these patients and a grand rounds lecture was provided to the ED providers. Outside of the ED, we have recently initiated a semi-urgent symptoms assessment clinic (SSAC) for oncology patients on systemic therapy, available during weekdays to serve as an alternate to the ED. At the SSAC, a patient is seen by an oncology nurse, and an internal medicine or oncology resident with the case discussed with the primary medical oncologist. We hope to repeat this analysis in the future to see if these interventions have led to a shorter “door-to-steroid” time and improved patient outcomes. Standardized work-up may provide the opportunity to develop an irAE predictive calculator, further improving upon quality of care for these patients. The Canadian Triage and Acuity Scale (CTAS) did not associate with prediction of irAE likelihood or grade in this study (data not shown).

Our study had several limitations. Currently, we only have access to data from the two EDs located in Kingston; however, patients at the CCSEO may present to one of the surrounding community EDs, where additional irAEs may have initially presented. Furthermore, some patients who presented to the ED for generalized weakness and were admitted for presumed progression of disease may have had an underlying irAE that was not identified, leading to an underestimate of the frequency of these toxicities presenting first in the ED. Other cancer centers may have different models of urgent care and oncology admission criteria, which may change the number of patients presenting to the ED as well as the frequency of these presentations being related to an irAE limiting the ability to generalize this study’s results.

## 5. Conclusions

Patients receiving ICIs have a high rate of ED utilization. Although the majority of visits are for reasons other than irAEs, those who are presenting with a new toxicity have high morbidity and risk for mortality. It is imperative that a systematic approach is applied to these patients and that ED providers are provided the toolkit to identify the patients who are receiving ICI as well as direction regarding initial work-up. This approach should be developed with the goal of increasing toxicity recognition and reducing the time to initiation of corticosteroid treatment.

## Figures and Tables

**Table 1:** Characteristics of patients receiving immune checkpoint inhibitors.

	Total ^a^
Patients With ED ^b^ Visit (% of total)	351 129 (37)
Gender Male (% of total)	198 (56)
Median Age (Range)	67 (19–91)
Melanoma (% of total) Adjuvant Metastatic	108 (31) 38 70
Lung Cancer (% of total) Consolidation Metastatic	172 (49) 22 150
Other (% of total) Renal Cell Urothelial/bladder	71 (20) 25 23
Doublet Regimen (% of total)	41 (12)
Developed an irAE ^c^ (% of total) New irAE in ED	111 (32) 17 (5)

^a^ Total of unique patients includes those treated from 2018 to 2019, ^b^ ED: emergency department; ^c^ irAE: immune-related adverse events.

**Table 2:** Characteristics of treatment and toxicity in patients who had first presentation of an immune-related adverse event at the emergency department.

ICI ^a^ Agent	Primary Tumour	# of Doses	Prior irAE ^b^	Presenting Symptoms	irAE Diagnosis	Admitted for irAE ^c^	Time to CS ^d^ (hrs)	30 Day ICU ^e^ Admission	Length of Hospitalization (Days)	Highest Grade ^f^	Was ICI Later Restarted
P ^g^	Melanoma	24	Yes	Fatigue	Arthritis	No	5			2	No
I ^h^/N ^i^	Melanoma	3	No	SOB/Rash	Pneumonitis	No	9			2	Yes
P	NSCLC ^l^	7	No	Nausea/Vomiting	Colitis	Yes	24	No	3	3	No
D ^j^/T ^k^	NSCLC	5	Yes	Weakness	Hypophysitis	Yes	4	No	10	3	No
I, P	Melanoma	2	Yes	Diarrhea	Colitis	Yes ^c^	49	No	7	3	No
P	Melanoma	3	Yes	Nausea/Weakness	Hypophysitis	Yes	43	No	14	3	Yes
I/N	Melanoma	2	No	Cough	Pneumonitis	Yes	25	No	2	3	No
P	Adj ^m^ Melanoma	1	No	Weakness	Myocarditis	Yes	36	Yes	13	5	
D	Adj NSCLC	1	No	Palpitations	Myocarditis	Yes	36	Yes	7	3	No
N	NSCLC	1	No	Fall	Encephalitis	Yes	269	Yes	32	4	No
P	NSCLC	2	No	Back Pain	Pneumonitis	No	240			1	Yes
P	NSCLC	1	Yes	Fever	Hepatitis	Yes ^c^	1 *	No	4	2	No
I/N	Melanoma	2	Yes	Back Pain	Neuritis	No	48			2	No
I/N	Melanoma	2	Yes	Fever	Arthritis	Yes ^c^	16	No	3	3	No
P	NSCLC	2	No	Weakness	PMR	No	24			3	No
P	Melanoma	1	No	Weakness, Abnormal Labs	Adrenalitis	Yes ^c^	83	Yes	12	5	
I/N	Melanoma	3	No	Headache	Hypophysitis	Yes	14	No	10	3	No

^a^ Immune checkpoint inhibitor, ^b^ Immune-related adverse events, ^c^ Admitted at next follow-up or upon second presentation to ED for the same symptoms, ^d^ ICU: Intensive care unit, ^e^ CS: Corticosteroid, ^f^ By Common Terminology for Adverse Events v5. ^g^ P: Pembrolizumab. ^h^ I: Ipilimumab, ^I^ N: Nivolumab, ^j^ D: Durvalumab, ^k^ T: Tremelimumab, ^l^ NSCLC: Non-small cell lung cancer, ^m^ Adjuvant. * Patient initially presented with acute infusion reaction treated immediately but was found days later to have hepatitis at follow-up.
